# Cardiac arrest secondary to arrhythmogenic right ventricular cardiomyopathy in an adolescent male

**DOI:** 10.1016/j.ipej.2022.06.001

**Published:** 2022-06-16

**Authors:** Meryam Jan, Michael S. Shillingford, Harma K. Turbendian, Sunita J. Ferns

**Affiliations:** a Wolfson Children's Hospital, Jacksonville, FL, USA; b University of Illinois College of Medicine, Peoria, IL, USA

## Abstract

Arrhythmogenic right ventricular cardiomyopathy (ARVC) is a rare, genetically-inherited cardiomyopathy that may be fatal. We present the case of a 17 year old male who presented after a witnessed cardiac arrest with indeterminate echocardiogram and electrocardiogram (ECG) findings for a specific etiology. Genetic testing revealed a mutation in the PKP2 and DSC2 genes, consistent with ARVC. This report outlines the presentation of ARVC as an aborted sudden cardiac death episode in a previously asymptomatic teenager, investigations for ARVC and highlights the importance of adequate cardiopulmonary resuscitation in the overall prognosis. Implantable cardiac defibrillator (ICD) placement for secondary prevention is necessary.

## Key points

• The first presentation of ARVC in the young can be a catastrophic arrhythmia.
• Initial ECGs and echocardiogram abnormalities can be subtle.
• Genetic screening for first degree relatives is important.
• First responder CPR plays an important role in the long-term prognosis.

## 1 Background

Arrhythmogenic right ventricular cardiomyopathy (ARVC) is a rare, genetic cardiomyopathy that results in replacement of myocardium of the right ventricle by fatty tissue [1]. Mutations in PKP2 are pathognomonic of the disease [2]. The disease may initially present as sudden cardiac death in adolescents and therefore it is important to identify family members with cardiac disease during initial evaluation. Although genetic testing is confirmatory, Electrocardiogram (ECG) and echocardiogram have significant roles in identifying this cardiomyopathy [3] however, as is evidenced in our case, arrhythmias may be the first presentation in young people before the appearance of characteristic ECG and imaging findings.

## 2 Case report

A 17 year old previously healthy male presented to the emergency department (ED) after a witnessed cardiopulmonary arrest in the middle of tennis practice. Cardiopulmonary resuscitation (CPR) was immediately initiated by his coach and emergency medical services arrived to the scene within 8 minutes. The time to the first shock was 10 minutes. He however continued to remain in ventricular fibrillation (VF) and received repeated shocks (6) with ongoing CPR for 60 minutes post arrest until establishing extracorporeal support. The patient had been completely asymptomatic leading up to the arrest. He had not been on medications and denied the use of stimulant drugs. Physical exam was unremarkable.

Family history revealed that his father was being followed by a cardiologist for premature ventricular contractions (PVC) since adolescence. Echo and Magnetic resonance imaging (MRI) results on the father were normal. The patient's paternal aunt had been diagnosed with arrhythmogenic right ventricular cardiomyopathy after an aborted cardiac arrest and is currently with an Implantable cardiac defibrillator (ICD).

**Fig. 1:**
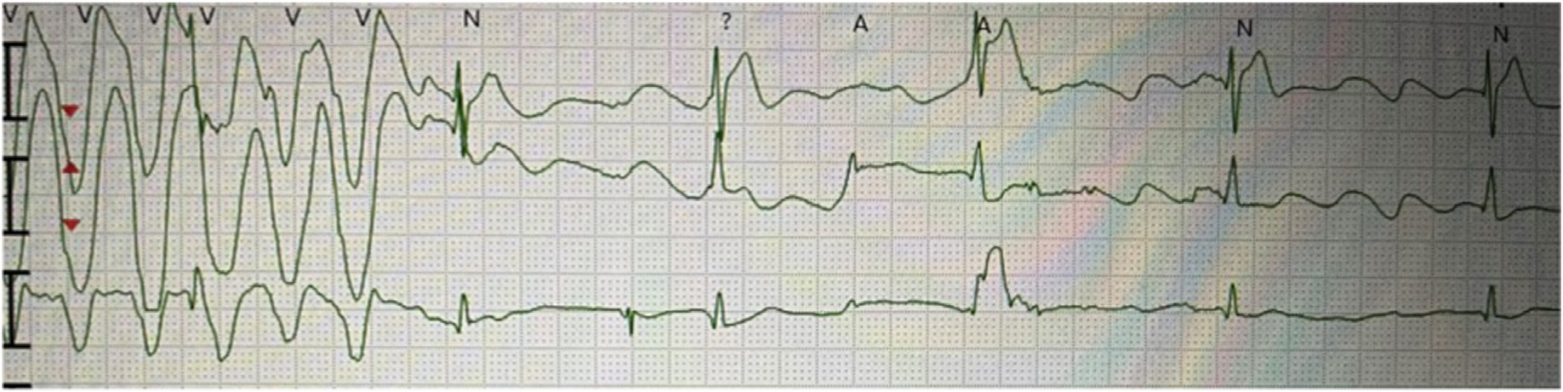
Polymorphic VT noted during transport of patient and recorded in between cardiac resuscitation efforts.

In the ED, the patient was in ventricular tachycardia/ventricular fibrillation (VT/VF) and advanced cardiac life support was continued. Once found to have a stable, perfusing ventricular rhythm, he was transferred to the cardiovascular intensive care unit (CVICU), but had repeated episodes of VT/VF requiring CPR and defibrillation during transport (Fig. 1). On arrival the patient was placed on an extracorporeal support device (Tandem Heart) while receiving amiodarone and lidocaine boluses for recurring VT/VF.

The patient was in a ventricular tachycardia with a left bundle branch block (LBBB) immediately post arrest. The ventricular tachycardia was incessant and required multiple antiarrhythmics. Once he cardioverted to sinus rhythm, initial QTc prolongation unto 503 msec was noted which normalized within the next few days.

**Fig. 2:**
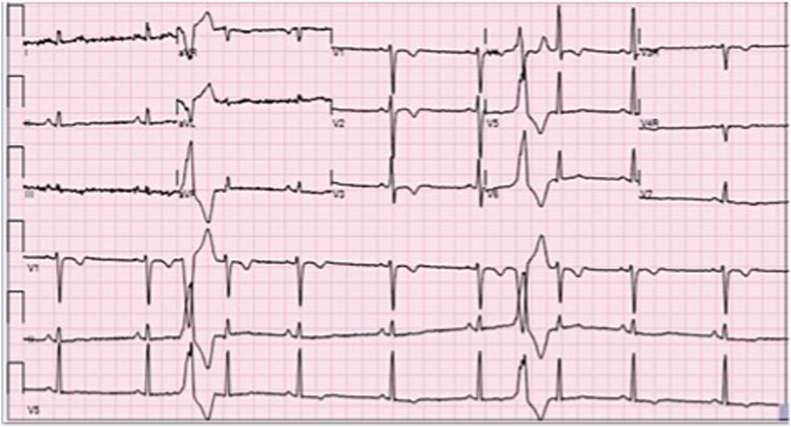
Abnormal inverted T waves noted across anterior precordial leads V1-3 and PVC morphology with a LBBB and inferior axis suggesting PVC origin from the anterior RVOT.

ECG demonstrated abnormal inverted T waves noted across anterior precordial leads and PVC morphology with a LBBB and inferior axis suggesting PVC origin from the anterior RVOT. Of note, there was no evidence of an epsilon wave which is a characteristic finding of ARVC in an older person (Fig. 2).

Initial transthoracic echocardiogram (TTE) showed severely decreased biventricular function. Structurally the heart was normal with normal coronary artery origins. Within the next few days the LV function normalized but the RV function continued to remain mildly depressed. There were no regional wall motion abnormalities noted.

Initial relevant investigations post cardiac arrest demonstrated a leukocytosis of 24 × 10^3^/μL with 85% neutrophils, hypokalemia of 2.4 mmol/L, hyperglycemia of 295 mg/dL, hypocalcemia of 6.6 mg/dL, and hypermagnesemia of 5.0 mmol/L. These laboratory findings were likely a result of ongoing CPR. The patient had lactic acidosis of 7.3 mmol/L suggestive of poor organ perfusion. Prothrombin time was elevated at 22 sec and fibrinogen was low at 161 mg/dL. Comprehensive urine toxicology screen was negative. An upper respiratory viral panel including COVID19 PCR and antibody test was unremarkable. Viral myocarditis panel was also negative.

Initial Troponin-I was 32.98 ng/mL which decreased to 0.28 ng/mL 13 days post-arrest.

**Fig. 3:**
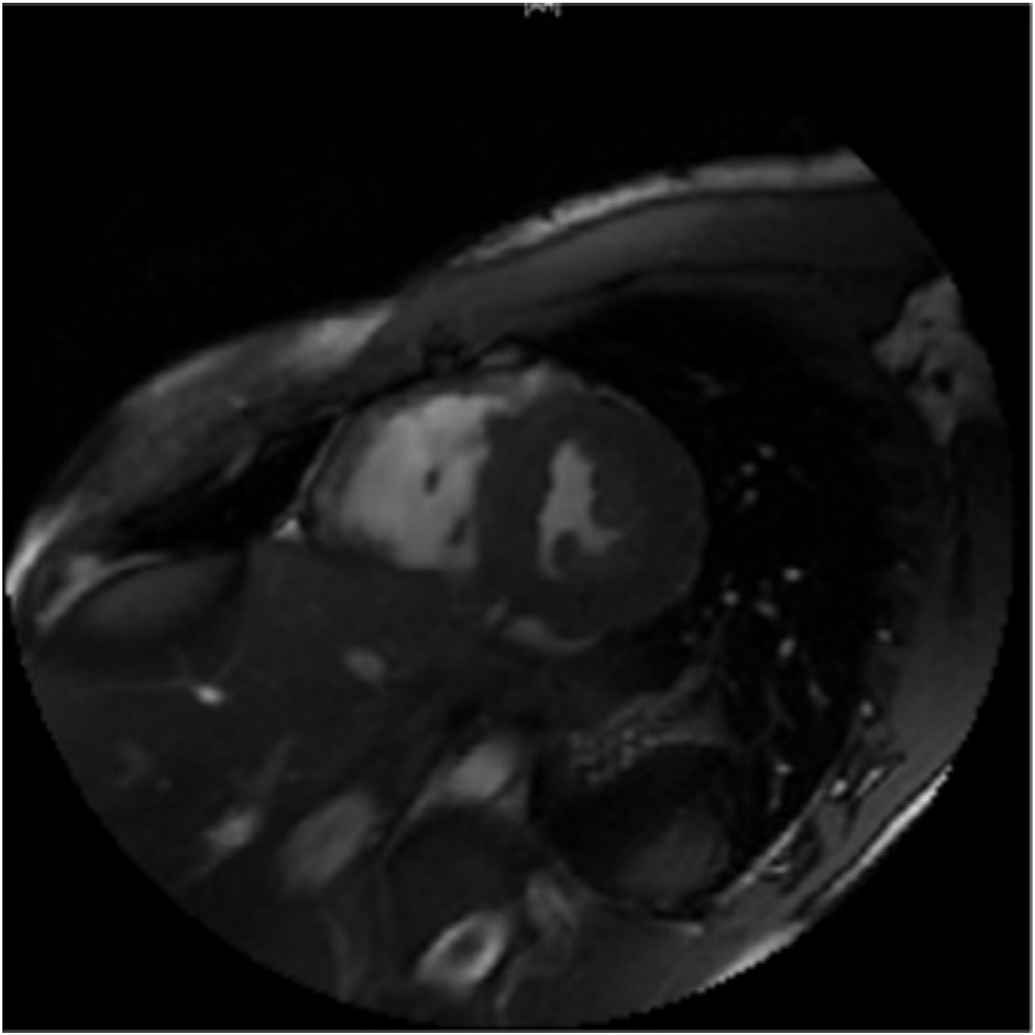
Transverse T2 gated MRI image depicting moderate right ventricular dilation with segmental wall thinning. In contrast the LV is normal in size with normal wall thickness.

Cardiac MRI was consistent with right ventricular (RV) EF of 40% and mild RV dilation with an indexed RV volume at 144 ml/m2. There was also noted to be RV hypokinesia of the free wall and inferior wall with segmental wall thinning and focal late enhancement (Fig. 3).

Brain MRI demonstrated areas of cortical infarct involving both frontal lobes, parietal lobes, and temporal lobes with associated cortical swelling, also within watershed areas. These findings were consistent with hypoxic ischemic encephalopathy (HIE). Electroencephalogram (EEG) did not reveal any subclinical seizures.

The patient remained on Tandem heart support for three days and received post-cardiac arrest care, included cooling with neuroprotective measures. The rhythm converted predominantly to normal sinus rhythm with occasional PVCs and the function gradually improved. He was separated from extracorporeal support and was extubated to room air without sequelae. A single-lead implanted cardioverter defibrillator (ICD) was placed for secondary prevention of cardiac arrest. He was discharged to an inpatient rehabilitation facility on amiodorone, mexiletine, and metoprolol tartrate. Over the next 3 months amiodarone was weaned and discontinued. Eight months later the patient continues to remain asymptomatic on mexilitine and metoprolol with only occasional PVCs noted on outpatient remote monitoring. Results of his most recent TTE showed a normal biventricular size and function with EF 76%.

**Fig. 4:**
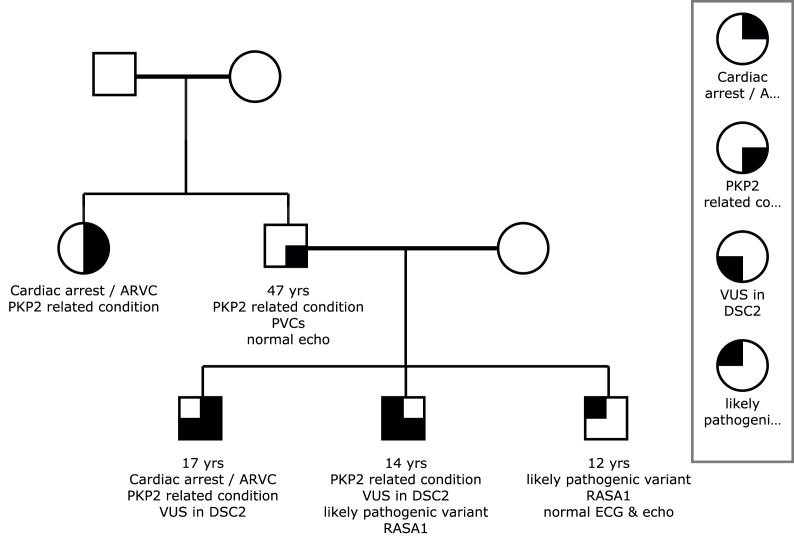
Three generation family tree denoting proband, parents and grandparents.

He demonstrated marked improvement in his neurological status with only mild memory deficits at follow up. Genetic testing was positive for a pathogenic variant in PKP2 and DSC2 (Invitae Clinical Genomics 2021). The patient's brother, father, paternal aunt, grandmother and first cousin also had genetic testing revealing the PKP2 mutation (Fig. 4).

Differential diagnosis included a genetic arrhythmia syndrome versus a cardiomyopathy. Myocarditis was low on the list of differentials in the absence of prodromal symptoms and biochemical markers. The cardiac MRI demonstrated a decreased RV EF of 40%, RV indexed volume of 144 ml/m2, and RV hypokinesia of the free wall and inferior wall. These met the three MR criterion for ARVC. Though ECG did not demonstrate classic findings of ARVC such as epsilon waves, inverted T-waves across V1–V3, and VT with a LBBB pattern indicating potential origin from the RV were noted. He had also experienced exercise-related cardiac arrest that may be seen in ARVC.

Catecholaminergic polymorphic ventricular tachycardia (CPVT) was considered as a possible etiology given that the patient's symptoms occurred during exercise and sympathetic surges exacerbate ventricular arrhythmias. Furthermore, the patient had an appropriate response to metoprolol for control of ventricular arrhythmia.

Long QT was also a possibility given the initial prolonged QTc of 500 ms. There were no prior ECGs for comparison. However, the patient's QTc improved throughout his hospital course, and in the setting of a recent cardiac arrest, ischemic brain injury and high doses of amiodarone, the initial prolonged QTc was likely not the underlying cause of the patient's cardiac arrest.

## 3 Discussion

ARVC is a cardiomyopathy involving fibrofatty replacement of the myocardium of the right ventricle [1]. Like other cardiomyopathies, this results in wall motion abnormalities that may result in decreased systolic function and subsequently cardiac arrest. The exact pathogenesis of the disease has not yet been identified, but has thought to be an interplay of factors including abnormalities of calcium channels and desmosomal proteins [4]. The most common genetic mutations involve the genes PKP2, DSG2, DSC2, DSP, and JUP [5].

Rare in the pediatric population, there is no established diagnostic guideline for ARVC in children [6] however diagnosis involves ECG, TTE, and genetic testing based on guidelines in adults [7]. TTE is the preferred imaging modality but may not always be diagnostic for ARVC as it may be limited in evaluation of the right ventricle due to its morphology and position in the chest wall [3]. Deshpande et al. [6] reviewed 16 cases of pediatric patients with ARVC of which five patients presented with cardiac arrest, six with ventricular tachycardia, and two with systolic heart failure. Nine of sixteen of their patients died from complications.

Proposed characteristic ECG findings in ARVC including epsilon waves in V1–V3 although it is quite rare [8,9]. Minor criteria, however, includes inverted T-waves in V1–V3, in patients over 12 years of age, in the absence of a right bundle branch block and sustained VT with LBBB [8,9]. Some of these findings, however, may not be pertinent in younger patients. For example, inversion of T-waves on ECG that may otherwise suggest repolarization abnormality are not an uncommon finding in children.

Our patient met minor diagnostic criteria by ECG and imaging modalities however it was ultimately genetic testing that confirmed the diagnosis of ARVC. This case underscores the importance of genetic testing in the young with a known family history of ARVC as routine diagnostic criterion established for adults may often be normal. Loss-of-function variants in PKP2 noted in our patient are known to be pathogenic for autosomal dominant arrhythmogenic right ventricular cardiomyopathy (ARVC). Additionally our patient also had a VUS in the DSC2 mutation which supports a correlation with autosomal recessive ARVC. Perhaps one of the most impressive outcomes this case illustrates is the importance of early and correct CPR. Despite resuscitation for over an hour, our patient had excellent physical and neurological outcomes. This in part, is due to early recognition of cardiac rhythms and cardiac arrest, prompt initiation of effective maintenance of oxygenation by CPR, and efficiently transporting the patient to a center that was able to provide a full spectrum of pediatric cardiac support.

## Funding

None.

## Declaration of competing interest

None.
